# Exceptional evolution of hepatocellular carcinoma

**DOI:** 10.11604/pamj.2019.34.53.18611

**Published:** 2019-09-26

**Authors:** Haithem Rejab, Aymen Trigui, Youssef Mejdoub, Hazem Ben Ameur, Ahmed Kchaou, Aida Jemal, Khayreddine Ben Mahfoudgh, Rafik Mzali

**Affiliations:** 1 Department of General and Digestive Surgery, Habib Bourguiba Hospital, Sfax 3027, Tunisia; 2 Department of Radiotherapy, Habib Bourguiba Hospital, Sfax 3027, Tunisia

**Keywords:** Hepatocellular carcinoma, spinal cord compression, radiotherapy

## Abstract

Hepatocellular carcinoma (HCC) tumor is the most common primary hepatic cancer. Bone metastases are rare with an incidence varying from 2% to 20% during autopsy. Spinal cord compression secondary to HCC is exceptional (0.03%-1.52%). It represents a therapeutic emergency. Therefore, it must be systematically searched in case of neurological signs. We report here two new cases of spinal cord compression secondary to HCC with a review of the literature.

## Introduction

Hepatocellular carcinoma (HCC) is the most common primary liver cancer. The most common metastatic sites are the lungs (37-70%) and lymph nodes (23-45%), bone involvement is rare. Spinal cord compression is exceptional. It represents less than 2%. We report here two new cases with review of the literature.

## Patient and observation

**Figure 1:**
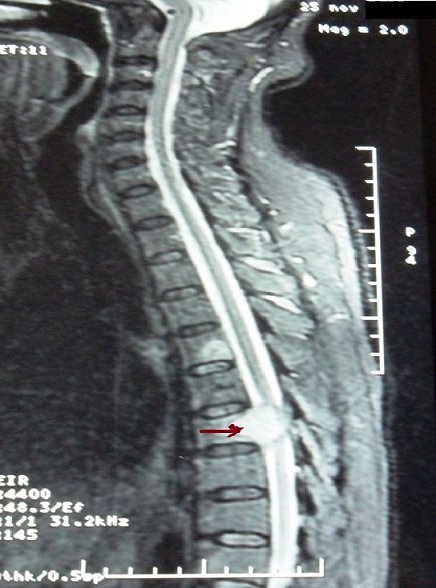
Spinal MRI, sagittal view: spinal cord compression in relation to the seventh dorsal vertebra

**Figure 2:**
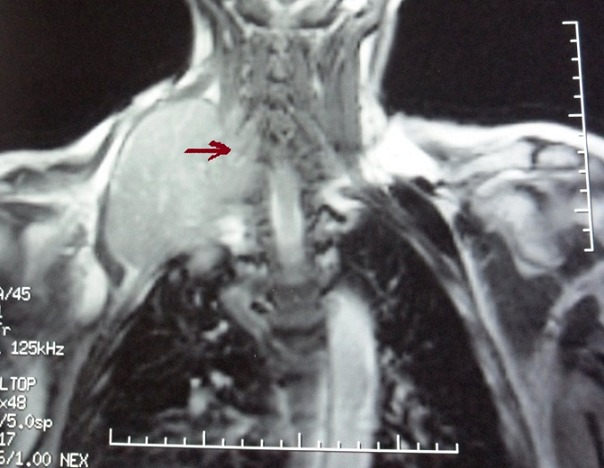
Spinal MRI, frontal view: a large right supraclavicular mass with dorsal extension at C7-D1

Case 1: we report the case of a 58-year-old smoker and alcoholic man bearer of chronic hepatitis B who was referred with sensorimotor deficit of right upper limb with epigastric pain. The radiograph of the right humerus was normal. Ultrasonography and abdominal computed tomography (CT) had concluded that the presence of hyper-vascularized hepatic lesions. The spinal magnetic resonance imaging (MRI) showed the presence of bone metastases in thoracolumbar spine and spinal cord compression in relation to the seventh dorsal vertebra (Figure 1). Biopsy of liver nodules under coelioscopy confirmed the diagnosis of hepatocellular carcinoma on cirrhotic liver. The patient had Child Pugh A. He had received a decompressive radiotherapy of bone metastases of the thoracic spine from D5 to D9 at a dose of 30Gy and was treated with steroids. A month later, the patient consulted for two masses in the right supraclavicular and the left base of the thorax with persistence of the sensorimotor deficit of right upper limb. Standard radiograph of the right humerus showed an osteolytic lesion with cortical break of the upper third of the right humerus. A new spinal MRI showed a large right supraclavicular mass with dorsal extension at C7-D1 (Figure 2), secondary spinal and rib lesions and posterior epiduritis at C7. The patient had an osteosynthesis of the right humerus with right cervico humeral radiotherapy at dose of 30Gy with improvement of symptoms. The patient is currently proposed for a targeted therapy with sorafenib and bisphosphonate.

**Figure 3:**
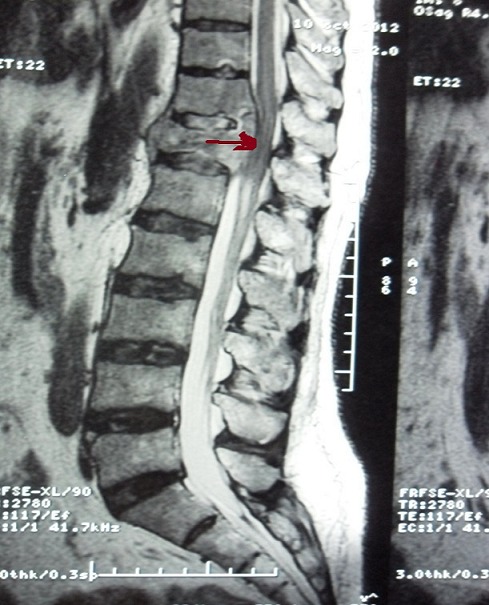
Spinal MRI, sagittal view: spinal cord compression in relation to the twelfth dorsal vertebra

Case 2: we report the case of a 62-year-old smoker and nonalcoholic man bearer of chronic hepatitis B with cirrhosis who had consulted for chronic diarrhea with weight loss. Abdominal ultrasonography showed a chronic liver disease with hyperechoic lesion of the segment V. Abdominal computed tomography showed hypodense lesions in the liver dome, segment V and VI. Hepatic MRI showed 3 hepatic lesions which evoked hepatocellular carcinoma. Biopsy of liver nodules confirmed the diagnosis of hepatocellular carcinoma on active cirrhotic liver. The patient had Child Pugh A. One month later, he consulted for spinal pain with motor deficit from a week ago. The spinal MRI showed the presence of bone metastases in thoracolumbar spine and spinal cord compression in relation to the twelfth dorsal vertebra (Figure 3). Bone scan showed increased uptake at the right trochonter and D5-7-12. Our patient had received a decompressive radiotherapy of bone metastases of the thoracic spine from D10 to L1 at a dose of 30Gy and was treated with steroids with mobility improvement. He is currently proposed for a targeted therapy with sorafenib and bisphosphonate.

## Discussion

Hepatocellular carcinoma (HCC) tumor is the most common primary hepatic. Incidence is steadily increasing and is currently the fifth cancer 1,12/100 000 according to data from cancer registries North Tunisia [1]. Bone metastases are rare with an incidence of only about 2%-20% during autopsy, while its clinical incidence varies between 5 and 7% according to different studies [2, 3]. The most frequent sites of the bone metastases are ribs, spine, femur, pelvis and humerus according to Khulman *et al*. Bone metastasis as the first presentation is rare. Kuhlman *et al.* and Liaw *et al.* have reported incidences of 3.3% and 5.1%, respectively [4]. The series by Okazaki *et al.* shows a higher incidence of 6.9%, which may be related to the small sample size, and only autopsy patients were included in the study [4]. We report here two cases of spinal cord compression secondary to HCC declaring the presence of bone metastases. In our first patient, spinal cord compression was the first presentation before the discovery of the diagnosis of HCC. In a review of cases spinal metastases causing a neurological syndrome of cord compression, HCC as the primary neoplasm was identified in less than 1% of cases (0.03% to 1.52%), while in a review series of HCC spinal cord compression has been reported in less than 2% making this presentation a very rare feature of HCC [5]. Our first patient had presented initially bone metastases in thoracolumbar spine and spinal cord compression in relation to the seventh dorsal vertebra. Then, he had had large right supraclavicular mass with dorsal extension at C7-D1 causing spinal epiduritis at C7 which is very rare. In our second case, our patient had spinal cord compression in relation to the twelfth dorsal vertebra secondary to bone metastases from dorsal vertebra. By reviewing the literature spinal cord compression typically arises from metastasis to one of three locations: the spinal column (in most cases), outgrowth of metastasis which eventually invades the epidural space; the paraspinal region through the neural foramen of vertebral bones, or rarely directly to the epidural space [6]. Our two patients had not pulmonary metastases so the vertebral venous plexus may be a route by which tumor cells of HCC spread to the bones and eventually to the epidural space.

In fact, metastatic spread to the spinal column usually occurs through the pulmonary circulation or the valveless vertebral venous system known as Batson's plexus [7]. Our patients, suffered from liver cirrhosis and HCC had developed not only extrahepatic metastases at an unusual site, but also had spinal cord compression as the initial manifestation of HCC in the first patient. Olubuyide *et al.* have reported that the pattern of metastases in HCC with cirrhosis is different from that in HCC without cirrhosis [8]. Indeed, HCC that arise in cirrhotic liver has few bony metastases [8]. Patients with bone metastases most often present with pain as the principal symptom. Very rarely, patient may present with bone pains without any symptom of underlying hepatic pathology, as seen in our first case. Apart from being most sensitive, cost-effective and noninvasive, MRI is also helpful in distinguishing between benign and malignant causes of spinal cord compression [9]. In all our patients, SCC was diagnosed with the help of MRI spine, which revealed the site of extradural cord compression with precision. Our patients had received a decompressive radiotherapy at a dose of 30Gy and were treated with steroids with mobility improvement. The treatment modalities available for spinal cord compression are individualized with a definite role of corticosteroids, radiotherapy and surgery. Corticosteroids help to relieve edema and to preserve neurological function. Radiotherapy is an important part of the management of spinal cord compression and it helps in pain relief, cytoreduction of tumor, prevention of progressive neurologic dysfunction and structural damage to the cord. It reduces pain in approximately 70%, improves motor function in 45% to 60%, and reverses paraplegia in 11% to 21% of the patients [10]. The outcome of radiotherapy is related to the neurological status prior to the treatment and the radiosensitivity of the tumor. It should also be given following surgery in patients who have not previously received radiation. The role of surgery in the treatment of spinal metastasis is often a subject of debate. The two main surgical approaches used for decompression are laminectomy with posterior fixation and anterior decompression of the spine with reconstruction [10].

## Conclusion

HCC should be included in the differential diagnosis of metastatic epidural spinal cord compression, because it may be the initial manifestation, with or without overt signs of liver disease.

## Competing interests

The authors declare no competing interests.
